# Analysis of state portrayals of the risks of e-cigarette use and the cause of the EVALI outbreak

**DOI:** 10.1186/s12954-022-00694-6

**Published:** 2022-10-05

**Authors:** Amanda Katchmar, Paul Shafer, Michael Siegel

**Affiliations:** 1 Department of Health Law, Policy, and Management, Boston University School of Public Health, 715 Albany St, Boston, MA 02118 USA; 2 Department of Public Health and Community Medicine, Tufts University School of Medicine, 136 Harrison Avenue, Boston, MA 02111 USA

**Keywords:** E-cigarette use, EVALI, Cigarette use, Messaging, State departments of public health

## Abstract

**Introduction:**

In August 2019, an outbreak of “e-cigarette or vaping product use-associated lung injury” (EVALI) prompted many states and health organizations to warn against the use of electronic cigarettes, or e-cigarettes, due to the presumed link between e-cigarette use and the illness. However, it was later shown that vitamin E acetate, a component of some illicit vaporizable THC products, was the causative agent in this outbreak.

**Methods:**

We conducted a series of cross-sectional surveys of the websites of all state departments of health to determine how they communicated the risk of e-cigarette use during and after the EVALI outbreak. We then paired this analysis with data from the 2016 through 2020 Behavioral Risk Factor Surveillance System to measure changes in cigarette and e-cigarette use.

**Results:**

Website data from 24 states was available for analysis at all three time points of interest, and BRFSS data was only available for 8 of these states. We found that by January 2020, a majority of the states surveyed did not list vaporizable THC use as a cause of EVALI; however, differences in state messaging did not appear to be associated with changes in e-cigarette and cigarette use.

**Conclusions:**

Given the number of states that did not appear to update their messaging regarding the cause of EVALI, we believe that states should re-evaluate this messaging to accurately communicate the risks of e-cigarette use.

**Supplementary Information:**

The online version contains supplementary material available at 10.1186/s12954-022-00694-6.

## Introduction

Electronic cigarettes, or “e-cigarettes,” are devices that vaporize nicotine-containing liquid for inhalation and were used by about 5% of American adults in 2020 [1, 2]. A 2021 Cochrane review indicated that e-cigarettes with nicotine are more effective than traditional nicotine replacement therapy for smoking cessation, and many Americans use e-cigarettes as smoking cessation aids; despite this, the majority of products are not approved by either the Food and Drug Administration (FDA) or the US Preventive Services Task Force (USPSTF) for such use [3, 4]. A 2014–2016 national survey of people who smoked cigarettes found that nearly one-quarter of those who attempted to quit smoking did so by switching to e-cigarettes, and a separate survey of adults in Montana who used e-cigarettes found that 56% of respondents did so to “quit or reduce cigarette use” [5, 6]. Because e-cigarettes simulate the act of smoking without delivering toxicants presented in tobacco smoke, these devices are generally regarded as a safer alternative to cigarettes, making them important for harm reduction [7]. However, e-cigarettes contain nicotine and potential carcinogens with unclear long-term health effects of their use [1].

In August 2019, there was an outbreak of what the US Centers for Disease Control and Prevention (CDC) called “e-cigarette or vaping product use-associated lung injury,” or “EVALI.” The illness was given this name because it was originally presumed to be related to e-cigarette use; all of the affected individuals reported vaping prior to becoming ill, with many reporting use of commonly-available e-cigarettes [8]. Over 2,800 individuals were sickened, many being under the age of 34, and sixty-eight individuals died [9]. Many of these patients experienced respiratory and gastrointestinal symptoms, such as cough, shortness of breath, nausea, and vomiting [10]. In response to the outbreak, many state and local governments levied or increased existing excise taxes on e-cigarettes [11]. Other states and localities, such as San Francisco and Massachusetts, issued bans on most e-cigarette purchasing [12, 13]. However, subsequent investigations by CDC, FDA, state health departments, and the cannabis industry determined that vitamin E acetate, a new thickening agent found in some illicit vaporizable THC products during the time period, to be the cause of the illness [14]. These products were considered to be “counterfeit” by the CDC and were commonly purchased from unregulated sources, such as the internet or “informal” sources such as friends and acquaintances [15].

Prior to the EVALI outbreak, surveys indicated that Americans viewed e-cigarette use as moderately risky, with about half believing that e-cigarettes were less harmful than combustible cigarettes [16, 17]. At the same time, there were concerns among public health professionals about the risks of e-cigarette use, the potential presence of carcinogens in vapor, and the youth appeal of the products [18]. While the initial public perception of e-cigarette use was somewhat favorable, there appeared to be an increase in the share of American adults who perceived e-cigarettes to be equally or more risky than combustible cigarettes between 2012 and 2017 [19]. This downturn in public opinion was believed to be due to regulatory actions taken by the United States government and media coverage of the risks associated with e-cigarette use [17, 20].

The linking of electronic cigarettes with EVALI—a serious and potentially acutely fatal respiratory illness—further led the public to believe that the devices carry the same or more risk than cigarette use. A poll conducted by the Kaiser Family Foundation in October 2019 indicated that only about 30% of respondents believed that e-cigarettes were safer than cigarette use [21]. Many news outlets did not appear to differentiate between nicotine-containing e-cigarettes and vaporizable THC products in their initial coverage of the EVALI outbreak and encouraged individuals to cease all e-cigarette use, not just illicit vaporizable THC use [22, 23]. News coverage of the outbreak in the United States had widespread influence on the public, even appearing to influence attitudes in England, where the proportion of individuals who believed that e-cigarettes were more risky than combustible cigarettes increased by one-third [24]. These findings imply that after the EVALI outbreak, messaging and media coverage led the public to view e-cigarette use as risky, equating the risk associated with these devices as being on par with or worse than that of smoking cigarettes.

Several studies have shown that cigarettes and e-cigarettes are economic substitutes, meaning that an increase in the price of one leads to an increase in the demand for the other [25–30]. For example, Cotti et al*.* utilized Nielsen retail scanner data to calculate that a 1% increase in the price of e-cigarettes increases the sale of cigarettes by 1.11% and that a nationwide e-cigarette tax proportional to the current tax on cigarettes would lead to a substitution rate of 5.5 packs of cigarettes purchased for every e-cigarette pod not purchased [26]. Similarly, Saffer et al*.* utilized data from the Current Population Survey Tobacco Use Supplements to measure the impact of Minnesota’s increased e-cigarette tax on smoking cessation. The authors estimated that a 10% rise in e-cigarette prices prompted a 13% increase in cigarette consumption and concluded that the tax increased adult smoking rates and reduced quit rates by 1.14% in the state, estimating that 32,400 additional adults who smoked cigarettes would have stopped smoking in the absence of the tax [29]. Our past analysis of consumer purchasing data indicates that cigarette sales increased immediately after the EVALI outbreak, suggesting that messaging that discouraged e-cigarette use may have prompted people who use e-cigarettes to switch to cigarettes [31]. Because the aforementioned studies focused on nationwide purchasing patterns, we hope to deepen our understanding of how messaging impacts e-cigarette purchasing by studying individual states’ responses to the outbreak.

A majority of the public believe that their state and local health departments provide reliable information about the health of people under their jurisdiction [32]. However, there has not been to our knowledge any research that explores how state departments of health (DOHs) approached the outbreak or portrayed e-cigarette use at the time of the EVALI outbreak. Further, no research has tracked portrayals of e-cigarette use by state DOHs over time and whether or not they updated their guidance with accurate information about the EVALI outbreak, or if changes in state messaging were correlated with changes in cigarette and e-cigarette use.

## Methods

This study follows the STROBE guidelines for cross-sectional studies [33].

### Analysis of messaging

To understand how state DOHs in the United States addressed e-cigarette use in regards to the EVALI outbreak, we reviewed messaging available from each department’s website at three time points to develop a longitudinal panel of state messaging regarding EVALI. Previous versions of the website, if available, were accessed through Archive.org. We searched these websites using the terms “EVALI,” “e-cigarettes,” “vaping,” “vaping illness,” “tobacco,” “cigarettes,” “smoking,” “THC,” and “vaporizable THC.” If information was not accessible on DOH websites, press releases and other media coverage was utilized to determine the state’s messaging at that time point, utilizing the same search terms along with “[State] Department of Health press release.” If a press release published on a state’s website during the time period of interest (e.g. between January 1st and January 31st of 2020), was identified, it was considered to be representative of the state’s messaging at that time point and included in our data analysis. If a press release was not identifiable for this time period, news stories (e.g. articles on a local newspaper or television news channel’s website) published during the time period of interest that included quotes or cited guidance from the state’s department of health were reviewed; messaging attributed to the state was considered to be representative of the state’s messaging at that time point.

One author (AK) coded multiple measures from each state DOH website and/or press releases based on a framework that was agreed upon by all authors prior to study commencement. Dichotomous measures included: (1) whether the website listed information about e-cigarette use and/or cigarette use, (2) whether it outlined the risks associated with e-cigarette and/or cigarette use, (3) whether it listed information about vaporizable THC use, (4) whether it outlined the risks associated with vaporizable THC use, (5) whether it listed information about EVALI, and (6) what it identified the cause of EVALI to be. These measures were coded at three time points: (1) mid-August of 2019, the peak of the EVALI outbreak; (2) January 2020, when it was shown that use of illicit vaporizable THC products was the causative agent in EVALI; and (3) September 2021, the time at which the analysis was taking place. If data was not available for a particular state at a particular time point, either due to the website not having been archived or information not being present on the current version of a website, it was noted in the dataset.

### Relationship between messaging and use

This state-year panel of messaging measures was paired with state-level prevalence of adult cigarette and e-cigarette use for 2016 through 2020. We obtained state-year adult cigarette use and e-cigarette use prevalence for 2016 to 2020 from the Behavioral Risk Factor Surveillance System (BRFSS), when available, supplemented by estimates from America’s Health Rankings and state reports in years when e-cigarette use was an optional item in BRFSS (2018 and 2019) [2, 34–50].

We then examined the relationship between changes in state-level smoking and vaping prevalence and the state DOH portrayals of e-cigarette use, using the measures hand-collected from our website analysis. Changes in cigarette and e-cigarette use were analyzed based on whether or not the state listed vaporizable THC use as a cause of EVALI via difference-in-difference analysis using the *xtdidregress* command in Stata, version 17 [51]. For this analysis, states that did not have website data for the first two time points (August 2019 and January 2020) were excluded, as were states that did not have BRFSS data regarding the prevalence of e-cigarette use for all years between 2016 and 2020.

We used a *p*-value of 0.05 to indicate statistical significance. Pre-intervention (i.e., before 2019, the start of the EVALI outbreak) trends were confirmed to be parallel using a parallel-trends test (Additional file 1: Figures S1 and S2), and no changes in anticipation of treatment were found using a Granger causality test.

## Results

### Analysis of messaging

The number of states whose websites were accessible for analysis varied between timepoints. All states were able to be analyzed for at least one time point for data regarding EVALI, but not all were available for every time point, as state department of health websites were either not saved on Archive.org at the time point of interest, press releases or news stories at that time point were not located, or, in the case of September 2021 (the date reflecting “current” messaging), did not appear to exist. The number of states with codable information regarding EVALI at each timepoint were as follows: 41 for August of 2019, 35 for January of 2020, and 44 for September 2021. Of these states, only 24 had data for all three time points (Additional file 1: Table S1).

**Fig. 1:**
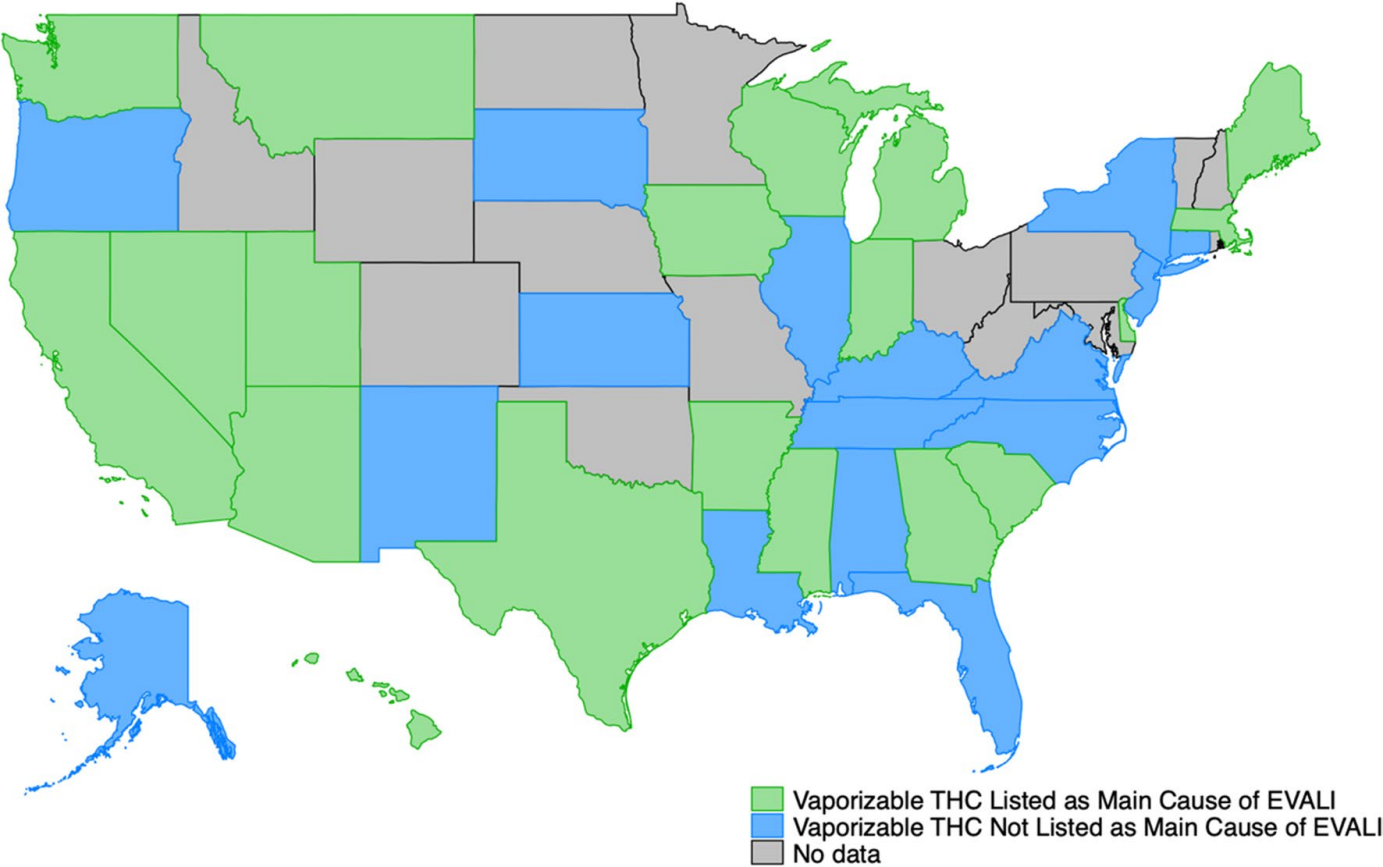
State messaging regarding vaporizable THC use as the main cause of EVALI, January 2020

Figure 1 depicts state messaging regarding the EVALI outbreak in January 2020 for all states that data was collected for; more specifically, it indicates whether or not the state listed vaporizable THC use, with or without vitamin E acetate, as the main cause of the outbreak. This timepoint is highlighted because it represents messaging from after it was known that vitamin E acetate, found only in illicit vaporizable THC products, was the cause of EVALI. By the January 2020, roughly half of the states that had available data listed vaporizable THC use as a cause of EVALI.

### Relationship between messaging and use

**Fig. 2:**
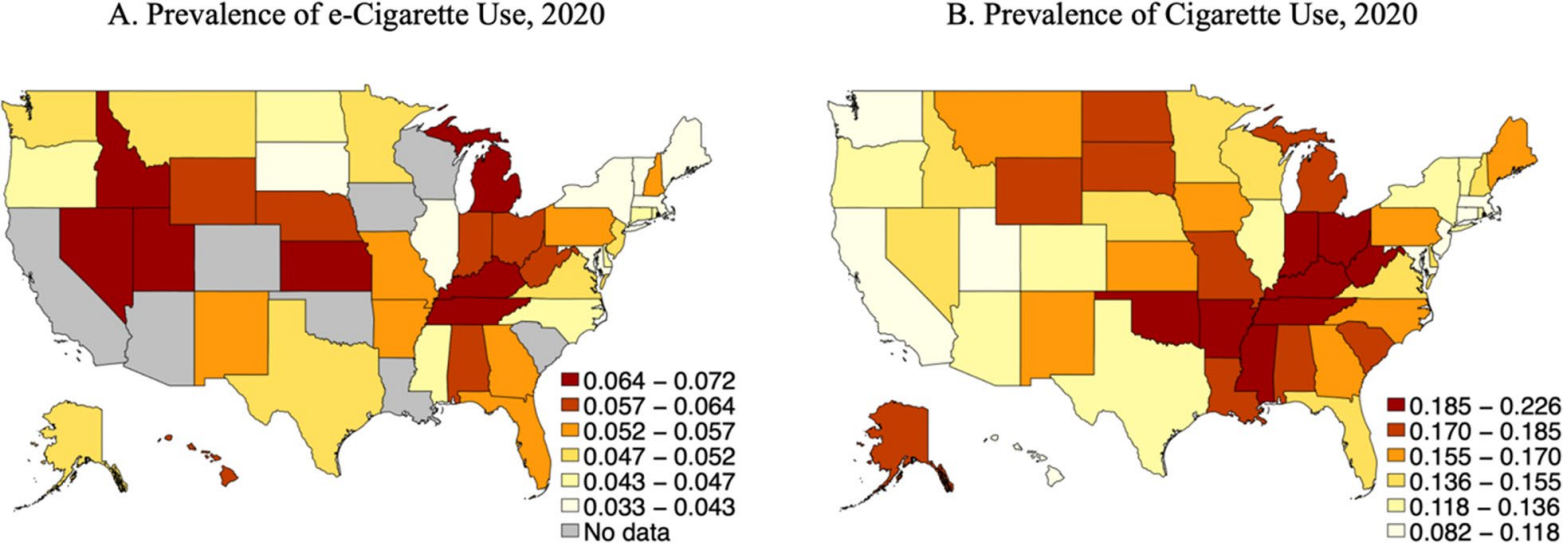
BRFSS prevalence data for e-cigarette and cigarette use, 2020

Figure 2 depicts the prevalence data for e-cigarette use and cigarette use in 2020. Availability of data on e-cigarette use varied by year; all states reported prevalence of e-cigarette use for 2016 and 2017, 36 did so for 2018, 15 did so for 2019, and 42 did so for 2020.

Because only 24 states had website data available for all three time points, only data from these states were included in Table 1, which lists different information that was coded for from each website. As of January 2020, only six states reported that vaporizable THC products were linked to EVALI, and only five states advised individuals to avoid vitamin E acetate. By September 2021, only 17 of the 24 states had listed vaporizable THC use as the main cause of EVALI, and fewer than half were advising individuals to avoid vitamin E acetate. The number of states listing vaporizable THC as the main cause of EVALI increased over time, as did the number of states asking individuals to avoid illicit vaporizable THC use and vitamin E acetate. However, a majority of states at each time point asked individuals to also avoid e-cigarette use in order to prevent EVALI (Table 1).

**Table 1:** Characteristics of State Department of Health Websites

Attribute	Time	Yes—n (%)	No—n (%)	Total
Lists Vaporizable THC as Main Cause of EVALI				
	August 2019	0 (0)	24 (100)	24
	January 2020	6 (25)	18 (75)	24
	Summer 2021	17 (71)	7 (29)	24
Notes that EVALI was caused by THC vaping products, not e-cigarettes				
	August 2019	0 (0)	24 (100)	24
	January 2020	14 (58)	10 (42)	24
	Summer 2021	18 (75)	6 (25)	24
Asks individuals to quit e-cigarette use to prevent EVALI				
	August 2019	12 (50)	12 (50)	24
	January 2020	15 (63)	9 (37)	24
	Summer 2021	23 (96)	1 (4)	24
Asks individuals to avoid using vaporizable THC to prevent EVALI				
	August 2019	10 (42)	14 (58)	24
	January 2020	15 (63)	9 (37)	24
	Summer 2021	20 (83)	4 (17)	24
Asks individuals to avoid vitamin E acetate				
	August 2019	0 (0)	24 (100)	24
	January 2020	5 (21)	19 (79)	24
	Summer 2021	11 (46)	13 (54)	24

For our regression analysis, we only considered states that had website data for the first two time points (August 2019 and January 2020). Of these states, only eight (CT, HI, KS, MA, NC, SD, TN, and UT) had BRFSS data regarding the prevalence of e-cigarette use for all years between 2016 and 2020.

The results of the difference-in-difference analyses are found in Table 2. The *p*-values for the parallel-trends tests were 0.2641 and 0.3508 for e-cigarettes and cigarettes, respectively, and the *p*-values for the Granger tests were 0.3065 and 0.3979, indicating confidence that pre-trends were parallel and there was no significant anticipation of treatment. The results from the difference-in-difference analysis indicate that there was not a significant difference in the change in e-cigarette use prevalence between states that listed vaporizable THC use as a cause of EVALI and those that did not (*p* = 0.783, Table 2). Similarly, there was no significant change in cigarette use prevalence after the EVALI outbreak between these groups (*p* = 0.409, Table 2).

**Table 2:** Difference-in-difference results for e-cigarette and cigarette use prevalence before and after EVALI outbreak

	Coefficient	SE	*t*	*p*	95% confidence interval	
E-cigarette use prevalence	0.0012089	0.0042248	0.29	0.783	− 0.0150984	0.0069206
Cigarette use prevalence	− 0.0040889	0.0046559	− 0.88	0.409	− 0.0087811	0. 0,111,988

## Discussion

In this paper, we analyzed state messaging related to the risks associated with e-cigarette and illicit vaporizable THC use following the EVALI outbreak and determined if there was a relationship between state messaging and e-cigarette and vaporizable THC use. We found that by January 2020, when it was understood that vitamin E acetate was linked to EVALI, only six of the 24 states analyzed reported that vaporizable THC products were linked to EVALI, and only five advised individuals to avoid vitamin E acetate. While we did not find any difference in cigarette or e-cigarette use prevalence between states that listed vaporizable THC use as a cause of EVALI and those that didn’t, messaging should be frequently updated to accurately reflect risks to the public and inform their health behaviors.

We had expected that cigarette use would increase in states that did not list illicit vaporizable THC use as a cause of EVALI based on past research that e-cigarette and cigarette use are substitutes, and that a decrease in the demand of e-cigarettes due to negative messaging would lead to an increase in smoking [25–30]. However, our findings did not fit this expectation, and we observed no association between state messaging and either e-cigarette or cigarette use within our study population and period. This lack of an observed association may be due to the waning public support of e-cigarette use prior to the EVALI outbreak. A number of studies had indicated that prior to the EVALI outbreak, the proportion of Americans who viewed e-cigarettes as less risky than cigarettes was decreasing, potentially due to increased media coverage of accidents involving e-cigarettes, increased regulatory activity, and literature suggesting that carcinogens were present in e-cigarette vapor [17, 20]. If a sizable share of the American public viewed e-cigarettes as equally or more risky than combustible cigarettes, as indicated by Huang et al*.*, any messaging about the risk posed by e-cigarette use in the context of the EVALI outbreak may not have contributed to any further change in attitudes or behaviors [19].

Additionally, the limited availability of data may have contributed to the lack of an observed correlation between state messaging and changes in use. Of all 50 states and the District of Columbia, only eight had both website and BRFSS data for the entire sample period, meaning our analysis may have been under-powered. We also only analyzed state DOH messaging because state websites are easily accessible, as opposed to temporary mass-messaging campaigns or messaging from advocacy groups, which may have reaches that are not contained within easily-distinguishable borders. Further, we based our analysis on the assumption that state DOH websites would be representative of other state-led mass media campaigns. However, state DOH websites may not have been where most individuals sought health information, especially in the pre-pandemic era, and they may not have accurately reflected greater trends in health messaging within each state. In future projects, it may be beneficial to utilize other methods to analyze state messaging.

Past analyses of the public’s risk perception regarding e-cigarette use have indicated that messaging, such as the information published on state DOH websites, may have contributed to a risk appraisal that was disproportionate to the actual risk that e-cigarette use carried. Dave et al., in an analysis of survey data collected before, during, and after the EVALI outbreak, found that immediately after the EVALI outbreak, the number of participants who believed that e-cigarettes were more harmful than cigarette use increased significantly. Further, as the CDC clarified the role of illicit vaporizable THC in the outbreak, risk perceptions only partially decreased [52]. Additionally, Viscusi analyzed the perceived risk of mortality from e-cigarette use among US adults and found that the perceived risk of e-cigarette use was much higher than the risk level generally agreed upon by the academic community. The author attributes this disparity to messaging campaigns not “distinguish[ing] any differential level of riskiness for conventional cigarettes and e-cigarettes” [53]. While our study indicates that changes in messaging did not lead to measurable changes in e-cigarette and cigarette use within our sample, findings from other studies indicate that they may have caused the public to assume that the risks associated with e-cigarette use were much higher than they were. Each state department of health maintains control over its own messaging, meaning that while the results of our analysis of the available data may not mirror the outcomes in states that we were unable to analyze, this analysis reveals a trend in which states may not have capacity to update all of their messaging to reflect evolving understanding and evidence.

As discussed above, our study has several limitations, including a small sample size and narrow scope of analysis. Future analyses could consider how state-sponsored messaging outside of DOH websites, such as public service announcements and mass-media campaigns, portrayed e-cigarette use-associated risk. Additionally, messaging from other advocacy groups, such as Parents Against Vaping and the Truth Initiative, has taken the same angle as early messaging from the CDC, suggesting that e-cigarette use is dangerous for youth and adults and should be avoided [54, 55]. Analyzing the effect of this messaging on perceptions and use may be a fruitful avenue for further research.

Given the findings from our study and other published works, we believe it is imperative that states re-evaluate their messaging regarding e-cigarette use and EVALI, not just to state that vitamin E acetate, and not e-cigarette use, was the cause of EVALI, but also to provide more clarity and accuracy in their messaging. E-cigarettes have been increasingly viewed as potential smoking cessation aids due to the lower amount of risk that they carry, compared to cigarettes. [7, 56] The FDA’s approval of select vaping products as smoking cessation devices is an example of this shift in viewpoint [57]. Given that the public’s assessed risk of e-cigarette use is much different than that of researchers and practitioners, a re-evaluation of messaging surrounding e-cigarette use and EVALI is needed if state health officials desire to reduce the use of combustible tobacco use.

## Conclusion

To understand how state DOH websites portrayed the risk associated with e-cigarette use during the EVALI outbreak and if those portrayals were associated with changes in cigarette and e-cigarette use, we analyzed state DOH websites at three time points: at the peak of the EVALI outbreak, after it was found that illicit vaporizable THC use caused the outbreak, and at the time of analysis. We found that by January 2020, three-quarters of the twenty-four states that we analyzed had listed that vaporizable THC products were not the main cause of EVALI, despite it being understood that vitamin E acetate, found in some illicit vaporizable THC products, was the causative agent in the outbreak. This messaging may have inadvertently portrayed e-cigarette use as the cause of the outbreak. We then analyzed BRFSS data to determine if there were changes in e-cigarette and cigarette use prevalence between states that listed illicit vaporizable THC as a cause of EVALI, and those that didn’t. While we did not find any significant changes in cigarette or e-cigarette use prevalence, past research has shown that messaging during this outbreak has shaped the public to view e-cigarette use as riskier than it actually is. Because of this, it is imperative that state DOHs update their messaging surrounding e-cigarette use to accurately reflect the risks associated with e-cigarette use.

## Supplementary Information

**Additional file 1**. This file includes results of a parallel-trends test and a table with the availability of state department of health websites at each time point.

## Data Availability

The website coding data utilized for this study are available from the corresponding author upon request; BRFSS data are publicly available at https://www.cdc.gov/brfss/data_documentation/index.htm.

## Abbreviations

BRFSS: Behavioral Risk Factor Surveillance System
CDC: United States Centers for Disease Control and Prevention
DOH: Department of Health
EVALI: E-cigarette or vaping product use-associated lung injury
FDA: United States Food and Drug Administration
THC: Tetrahydrocannabinol
USPSTF: United States Preventive Services Task Force
