# Supplementary material for: Analysis of state portrayals of the risks of e-cigarette use and the cause of the EVALI outbreak
Source: Harm Reduct J. 2022 Oct 5;19:112. doi: 10.1186/s12954-022-00694-6 (PMC9535934; doi:10.1186/s12954-022-00694-6)
Supplement: Supplementary file 1 — Additional file 1. This file includes results of a parallel-trends test and a table with the availability of state department of health websites at each time point. [file 12954_2022_694_MOESM1_ESM.docx]

**SUPPLEMENTAL MATERIAL**

**
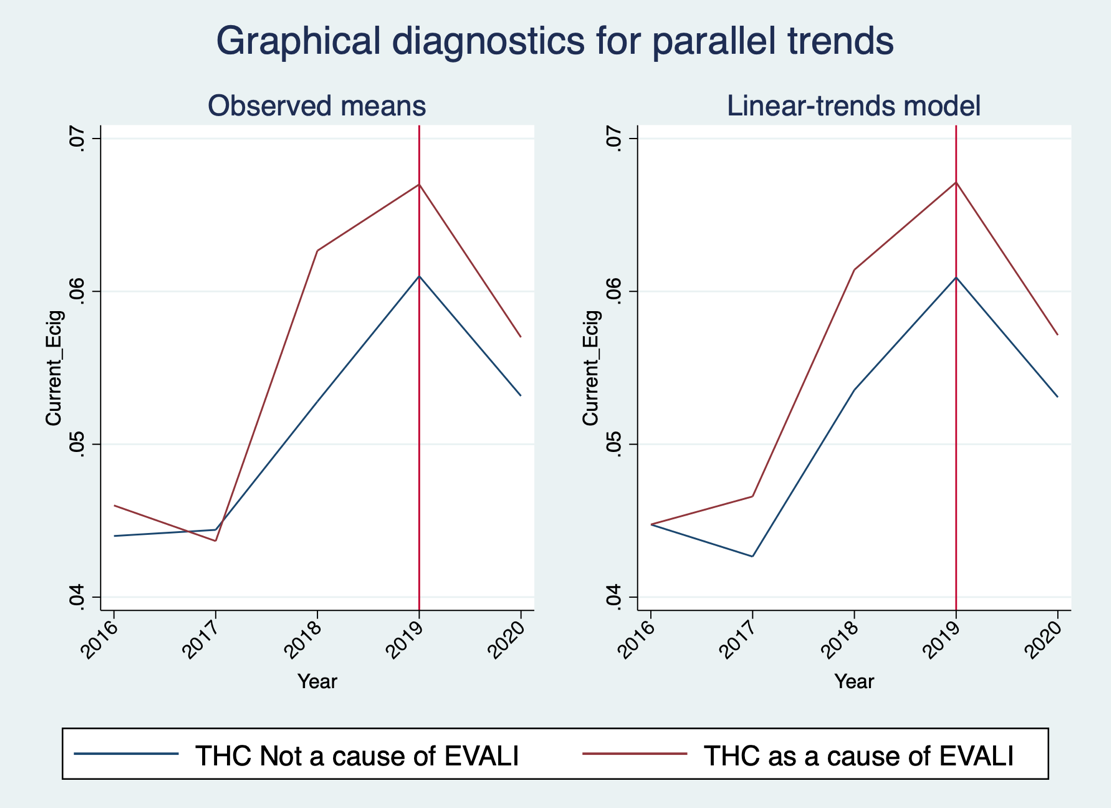
**

**Figure S1.** Graphical Confirmation of Parallel Trends, E-Cigarette Use


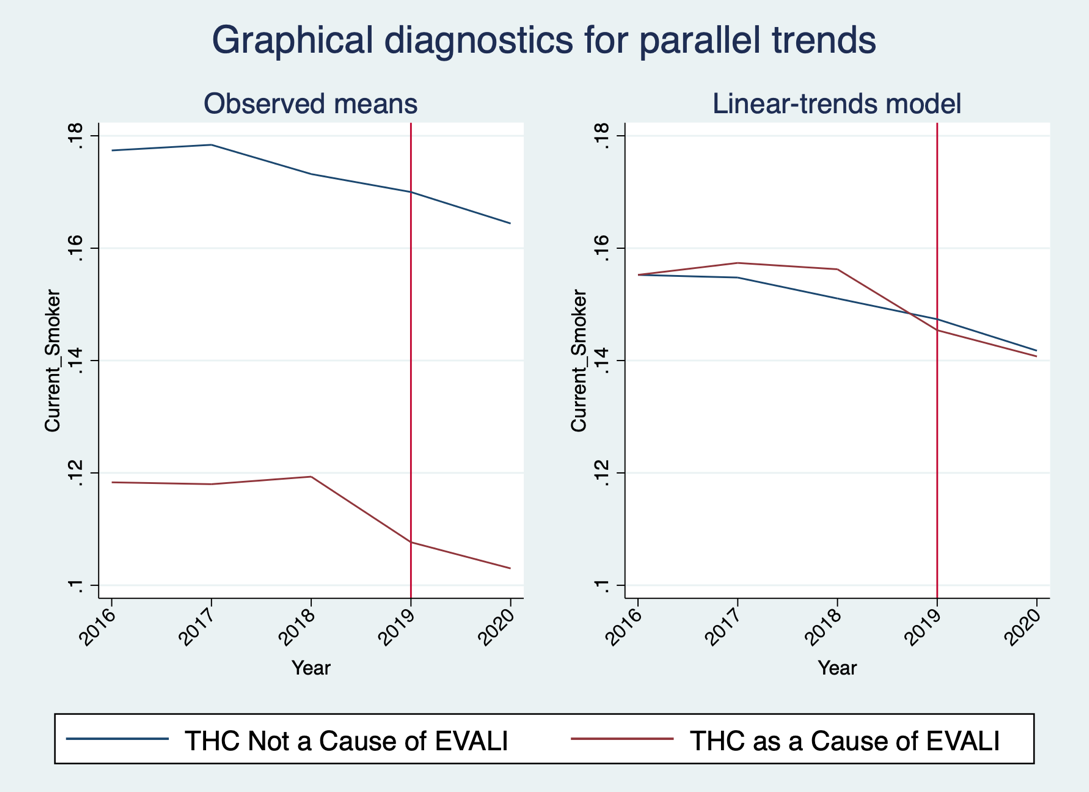


**Figure S2.** Graphical Confirmation of Parallel Trends, Cigarette Use

**Table S1.** Availability of EVALI Information at Each Time Point

| State | August 2019 | January 2020 | September 2021 | All Three? |
| --- | --- | --- | --- | --- |
| Alabama | No | Yes | Yes |  |
| Alaska | Not archived | Yes | No |  |
| Arizona | Yes | Yes | Yes | Yes |
| Arkansas | No | News Article | Yes |  |
| California | Yes | Yes | Yes | Yes |
| Colorado | Yes | Not Archived | Yes |  |
| Connecticut | Yes | Yes | Yes | Yes |
| Delaware | No | Yes | Yes |  |
| DC | Yes | Not Archived | Yes |  |
| Florida | No | Yes | Yes |  |
| Georgia | Yes | Yes | Yes | Yes |
| Hawaii | Yes | News Article | Yes | Yes |
| Idaho | News Article | Not Archived | No |  |
| Illinois | Yes | Yes | Yes | Yes |
| Indiana | News Article | News Article | Yes | Yes |
| Iowa | Yes | Yes | Yes | Yes |
| Kansas | Yes | Yes | No |  |
| Kentucky | News Article | News Article | Yes | Yes |
| Louisiana | Yes | Yes | Yes | Yes |
| Maine | Yes | Yes | Yes | Yes |
| Maryland | Yes | Not Archived | Yes |  |
| Massachusetts | Yes | News Article | Yes | Yes |
| Michigan | No | Yes | Yes |  |
| Minnesota | Yes | Not Archived | Yes |  |
| Mississippi | Yes | Yes | Yes | Yes |
| Missouri | Yes | Not Archived | Yes |  |
| Montana | Yes | Yes | Yes | Yes |
| Nebraska | Yes | Not Archived | Yes |  |
| Nevada | News Article | News Article | No |  |
| New Hampshire | No | Not Archived | Yes |  |
| New Jersey | Yes | Yes | Yes | Yes |
| New Mexico | No | Yes | Yes |  |
| New York | Yes | Yes | Yes | Yes |
| North Carolina | Yes | Yes | Yes | Yes |
| North Dakota | Yes | Not Archived | Yes |  |
| Ohio | Yes | Not Archived | Yes |  |
| Oklahoma | Yes | Not Archived | Yes |  |
| Oregon | Yes | Yes | Yes | Yes |
| Pennsylvania | Yes | Not Archived | No |  |
| Rhode Island | Yes | Not Archived | No |  |
| South Carolina | No | Yes | Yes |  |
| South Dakota | News Article | Yes | Yes | Yes |
| Tennessee | Yes | Yes | Yes | Yes |
| Texas | Yes | News Article | Yes | Yes |
| Utah | Yes | Yes | Yes | Yes |
| Vermont | Yes | Not Archived | Yes |  |
| Virginia | Yes | Yes | Yes | Yes |
| Washington | Yes | Yes | Yes | Yes |
| West Virginia | News Article | Not Archived | No |  |
| Wisconsin | No | Yes | Yes |  |
| Wyoming | Yes | Not Archived | Yes |  |
